# Evaluation of Bacterial Load and Antibiotic Resistance Pattern of *Staphylococcus aureus* from Ready-to-Eat Raw Beef in Bahir Dar City, Ethiopia

**DOI:** 10.1155/2021/5560596

**Published:** 2021-03-25

**Authors:** Bizuneh Tsehayneh, Taddesse Yayeh, Birhan Agmas

**Affiliations:** Department of Veterinary Science, College of Agriculture and Environmental Sciences, Bahir Dar University, Bahir Dar, Ethiopia

## Abstract

**Background:**

S*taphylococcus aureus* is one of the most important causes of food-borne intoxication and the most frequent antibiotic-resistant pathogen in the world. Regular evaluation of the current safety status of food is a proactive measure to minimize the possible danger of food-borne pathogens. Therefore, this study was conducted to assess the bacterial load and antibiotic resistance profile of *S. aureus* from ready-to-eat raw beef in Bahir Dar city, Ethiopia. *Methodology*. This cross-sectional study was conducted from October 2018 to April 2019 by collecting a total of 101 raw beef samples from butcher shops using a simple random sampling method. Isolation and microbial load determination of *S. aureus* use were performed by conventional culture method and an antibiotic susceptibility test was conducted by using Kirby Bauer disk diffusion method on the Mueller–Hinton agar. The data were analyzed by using STATA software version 12.0.

**Result:**

Out of 101 raw beef samples, 55 (54.45%) were positive for *S. aureus* with a mean bacterial count of 3.40 ± 0.63 (log_10_ cfu/g). About 13% of butcher shops had unacceptable and potentially dangerous (above 10^4^ cfu/g) bacterial load. High *S. aureus* drug resistance was observed on penicillin (92.73%) followed by cefoxitin (74.5%), tetracycline (63.63%), and clindamycin (50.9%). On the other hand, there was the highest susceptibility for ciprofloxacin (100%) followed by gentamycin (90.91%) and erythromycin (87.27%). Multidrug resistance was also found in 54 (98%) of the isolates.

**Conclusion:**

In this study highly drug-resistant *S. aureus* was incriminated as the main meat contaminant in butcheries of Bahir Dar city. Therefore, appropriate antimicrobial use and staphylococcal control methods should be employed to prevent *S. aureus* intoxications in foods.

## 1. Background

Animal origin food items are rich sources of nutrients and provide a variety of micronutrients to humans that are not obtained in plant-derived foods [1]. Meat is one of the most nutritive and favorite animal-source foods. Due to its high water content (0.99 water activity) and being rich in proteins, minerals, and other nutrients which are suitable for microbial growth, meat is a highly perishable food that can cause infection in humans and also can lead to economic loss due to spoilage [2, 3].

A large number of food-borne zoonotic diseases often occur due to the consumption of contaminated animal origin foods such as meat and milk [4]. The common food-borne pathogens which are responsible for most of the food-borne disease outbreaks are *Listeria monocytogenes, Escherichia coli* O157 : H7, *Staphylococcus aureus, Salmonella enterica, Bacillus cereus, Vibrio* spp., *Campylobacter jejuni, Clostridium perfringens*, and Shiga toxin-producing *Escherichia coli* (STEC) [5]. Among the bacteria predominantly involved in food-borne diseases (FBD), *S. aureus* is one of the leading causes of food-borne intoxication throughout the world resulting from the consumption of preformed staphylococcal enterotoxins [6–8]. Due to poor hygienic practices and a low level of awareness, this problem is worse in developing countries.

*Staphylococcus aureus* is a food-borne pathogen which is responsible for contamination of different food products and results in food spoilage and reduction of food safety and shelf life and causes food-borne poisoning via the production of deadly enterotoxins [9], but other enterotoxigenic coagulase-positive staphylococci such as *S. hyicus* and *S. intermedius* have also been implicated in staphylococcal food poisoning [10]. It is a frequent component of the skin flora of food animals and therefore can be expected to be present on raw meats and other meat products [11].

Staphylococcal food-borne intoxication (SFI) is often associated with the ingestion of highly heat-stable staphylococcal enterotoxins [12]. The acceptable level of *S. aureus* in ready-to-eat food should be below 10^3^ colony-forming units per gram (cfu/g) of food. If the amount of bacteria is greater than 10^4^ cfu/g, the food is unsatisfactory and potentially hazardous for health and/or unfit for human consumption [13]. Ingestion of nanogram to a microgram of staphylococcal enterotoxin contaminated food can cause serious illness ranging from minor skin infection to life-threatening diseases [14].

Antimicrobial resistance is a serious threat to public health across the globe. A wide variety of antimicrobial drugs are employed to treat *S. aureus* infections [15]. However, the emergence and spread of antimicrobial-resistant *S. aureus* isolates constitute a global challenge for the effective treatment and control of these infections [16–18].

Even though effective food safety systems are vital to maintain consumer confidence in the food system and to provide a sound regulatory foundation for domestic and international trade in food, there are gaps in the Ethiopian food safety system on legal and policy framework, food-borne diseases surveillance, coordination of organizations involved in food safety management, and laboratory services for relevant food hazards [19]. Poor food handling and sanitation practices, inadequate food safety laws, weak regulatory systems, lack of financial resources, and awareness about proper food handling create a conducive environment for the spread of food-borne and food poisoning etiologic agents in Ethiopia [20]. The widespread habit of raw meat in the form of simple cut strips of meat which is locally called “Kurt” and minced meat (Kitfo) in the population is suggestive of the risk of food-borne bacteria including *S. aureus* [21, 22]. Moreover, raw beef is available in open-air local butchers of Ethiopia without the cold-chain process which could be serving as a potential source for food-borne illnesses [23, 24].

Besides the prevalence of *S. aureus* in meat reaches up to 40% [25] in butcher shops of Mekelle city Ethiopia, the burden and public health impact of food-borne illness related to *S. aureus* infection are poorly understood [21]. Moreover, there is a paucity of data regarding *S. aureus* from ready-to-eat raw beef in Bahir Dar city. Therefore, this study was focused on isolation and identification of *S. aureus,* measuring its magnitude and antibiotic resistance pattern from ready-to-eat raw beef to provide useful information regarding staphylococcal loads and their antibiotic resistance profile in Bahir Dar city, Ethiopia.

## 2. Materials and Methods

### 2.1. Media and Reagents

The main materials, media, and reagents used in this study are listed as follows: icebox (Cello Chiller Ice Pack, India), Stomacher (Stomacher® 400 Circulator, UK), sterile stomacher plastic bags (Stomacher® 400 circulator bags, UK), peptone water (Difco, USA), Gram Stain Kit (HiMedia, India), purple agar base (Difco, US), mannitol salt agar (Oxoid, UK), blood agar base (BBL, USA), Mueller-Hinton agar (HiMedia, India), nutrient agar (Oxoid, UK), rabbit plasma (NVI, Ethiopia), H_2_O_2_ (HiMedia, India), and O/F medium (HiMedia, India).

### 2.2. Study Area and Study Design

The current cross-sectional study was conducted from October 2018 to April 2019 in Bahir Dar city (located 565 km north-northwest of Addis Ababa) in ready-to-eat raw beef retailers of butcheries. Geographically, Bahir Dar is located between 11.29° to 11.38° north latitude and 37.23° to 37.36° east longitude. Its average elevation is estimated to be 1810 meters above sea level. The city's mean annual temperature ranges from 7.1°C to 29.7°C, whereas the annual mean temperature was 20.85°C [26].

### 2.3. Sample Size Determination and Sampling Procedures

In Bahir Dar city, about 137 licensed butcher shops were operating on meat and meat products, and all the butcheries were included in the sampling procedure. The lists of all butcher shops were obtained from the health centers. The sample size was calculated by using the Thrusfield formula for a small sampling population [27] and a total of 101 retailers were selected based on simple random sampling.

### 2.4. Data Collection

From randomly selected butcher shops, about 250 grams of ready-to-eat raw beef (Kurt) samples were collected in sterile stomacher plastic bags and kept in an icebox containing ice. The collected samples were immediately taken to Bahir Dar University, Institute of Technology, food microbiology laboratory, for homogenization and the homogenates were transported to the Amhara Public Health Institute (APHI), microbiology laboratory unit, within 4 hours by keeping the cold chain for bacteriological analysis.

### 2.5. Bacteriological Investigation

Isolation and identification of *S. aureus* were done according to the methods described by [9, 28]. Briefly, 25 grams of raw beef sample was transferred aseptically into a sterile stomacher bag containing 225 ml of peptone water and homogenized for 3 minutes using a stomacher. From the homogenate, a loopful aliquot was spread on blood agar plates (5% defibrinated sheep blood) for growth and hemolytic pattern of *S. aureus* and on mannitol salt agar (MSA) for the growth of golden yellow colonies and incubated from 24 to 48 hours at 37°C. A loopful of a pure culture of presumptive colonies from each sample was streaked on nutrient agar and incubated for 24–36h at 37°C for gram stain and further biochemical tests (catalase, coagulase test, and oxidation–fermentation test). In addition, a representative colony was also inoculated and incubated aerobically at 37°C for 24 hours on purple agar base media (with 1% maltose) for rapid fermentation of maltose by *S. aureus* resulting in yellow discoloration of the medium due to acidic metabolic product of maltose. Uninoculated media was incubated as a negative control to check for sterility.

Gram-positive colonies with beta-hemolysis on blood agar, golden yellow color on MSA, catalase and coagulase positive, fermentative on oxidation-fermentation test, and yellowish discoloration of purple agar base medium were confirmed as *S. aureus* [9].

### 2.6. Enumeration of *Staphylococcus aureus* Count

In addition to identification, microbial counts of *S. aureus* were conducted on MSA by using the spread plate count method. To enumerate *S. aureus* in meat samples, tenfold serial dilutions from the original homogenate were prepared and a 0.1 ml sample from serial dilutions was spread on MSA and incubated from 24 to 48 hours at 37°C. Presumptive colonies were undergoing confirmatory test and golden yellow colonies on MSA with catalase and coagulase-positive isolates, complete hemolysis on blood agar, and rapid fermentation of maltose on purple agar base media were identified as *S. aureus* count and the number of cfu/g of the test sample was calculated by the following formula [29, 30]:

(1) $$\begin{array}{c} \text{cfu per gram of sample}=\frac{C}{d*v}, \end{array}$$

where *c* is the number of colonies on the counted plate, *d* is the dilution rate of the counted plate, and *v* is the inoculated volume of this dilution.

### 2.7. Antimicrobial Susceptibility Testing

All positive isolates of *S. aureus* were subjected to antibiotic susceptibility test by using the Kirby Bauer disc diffusion method as per Clinical Laboratory Standards Institute (CLSI) of USA guidelines on Mueller-Hinton agar (MHA). The antibiotics were selected based on the groupings of antibiotic agents with the United States Food and Drug Administration clinical indications that should be considered for routine testing and reporting on nonfastidious organisms. One representative antibiotic agent from each subclass of antibiotics groups, commonly used and most available antibiotics for the treatment of staphylococcal-related diseases in animals and humans, was selected. Based on the above criteria, 9 antibiotics [chloramphenicol (30 *µ*g), ciprofloxacin (5 *µ*g), cefoxitin (30 *µ*g), clindamycin (2 *µ*g), erythromycin (15 *µ*g), gentamycin (10 *µ*g), penicillin (10 units), tetracycline (30 *µ*g), and trimethoprim-sulfamethoxazole (1.25/23.75 *µ*g)] were selected for this study [31].

For the susceptibility test, three to five well-isolated colonies of the same morphological type were selected from nutrient agar plate culture and transferred into test tubes containing sterile saline and mixed thoroughly. The density of the suspension was adjusted to McFarland 0.5 by the addition of saline or more *S. aureus* colony. A sterile swab was dipped into the suspension and the excess of inoculums were removed by pressing it against the sides of the tube to avoid overinoculation of plates. The inoculums were spread evenly over the entire surface of the agar plate by swabbing in three directions. Antibiotic discs were applied firmly on the agar surface and incubated for 24h at 37°C. The diameter of the zone of inhibition around the disc was measured using a ruler in millimeter (mm) and interpreted according to the standard of CLSI as susceptible, intermediate, or resistant [31–33]. Those isolates showing resistance to three or more antibiotics were considered as multiple drug resistant (MDR) [9].

### 2.8. Data Quality Assurance

The data quality and the reliability of the study findings were assured by following standard operating procedures and the routine use of control bacterial strains. The sterility of prepared media was checked by incubating some randomly selected plates for 24–48 hours at 37°C. Uninoculated media was incubated as a negative control to check for sterility. The quality of the culture media and test procedures were thoroughly checked using standard American Type Culture Collection (ATCC) strain of *S. aureus* (ATCC25923) as a positive control for screening tests, confirmatory tests, and disk diffusion antibiotic susceptibility tests. *Escherichia coli* ATCC-25922 was used as a negative control for culture on mannitol salt agar.

### 2.9. Data Management and Statistical Analysis

Raw data and laboratory findings were encoded into Microsoft Excel, exported into STATA software, version 12.0, and analyzed using descriptive statistics such as frequency, percentages, and mean and standard deviation (SD). In all the analyses, the confidence level was held at 95% and *P* value was assumed less than 5% (*P* < 0.05).

## 3. Results and Discussion

### 3.1. Isolation Rate of *Staphylococcus aureus*

In the present investigation, from a total of 101 ready-to-eat raw beef samples subjected for cultural and biochemical isolation, 55 (54.45%) were positive for *S. aureus* which suggests that the bacteria could be one of the major food contaminants in butcher's shops of Bahir Dar city.

The present result was higher than the previous findings of 29.17% from butcher shops of Addis Ababa [34]; 32.22% from retail houses of Jigjiga town [35]; 40% from hand and knife in butcher shops of Mekelle city [25]; and 36.5% from ready-to-eat meat in Debre-Zeit, Ethiopia [36]. These differences in prevalence may reflect the level of meat contamination during their food handling practices, level of environmental hygiene, and the degree of awareness related to microbial contamination. This notion was supported by Kibrom [34] who stated that a high level of food contamination with *S. aureus* has been related to improper personal hygiene of employees during food handling and processing.

### 3.2. Microbial Load of *Staphylococcus aureus*

**Figure 1:**
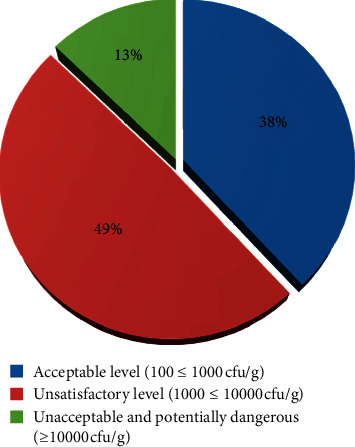
Microbial quality levels of butcher shops based on *S. aureus* count from raw beef in Bahir Dar city.

The enumeration of *S. aureus* was performed on MSA using the spread plate technique. The minimum and maximum *S. aureus* counts in ready-to-eat raw beef samples of Bahir Dar city were 2.48 and 5.08 (log_10_ cfu/g), respectively, with a mean and standard deviation of 3.40 ± 0.63 (log_10_ cfu/g). Based on CFS (2014) microbiological guidelines for food, none of the butcher shops had a good microbial level (below 100 cfu/g) and the majority of them (49%) had unsatisfactory levels followed by an acceptable microbial level (38%) for *S. aureus* counts (Figure 1). The mean count of *S. aureus* in this study was in line with 3.88 log_10_ cfu/g reported from fresh meat Bahir Dar City [37]. About 13% of butcher shops had an unacceptable and potentially dangerous bacterial load. If the bacterial count exceeds the above standard in fresh meat, then the meat is not acceptable and this indicates alarm signals on meat hygiene along the meat chain from the abattoir to butcher shops [38].

The mean count of *S. aureus* in this study was lower than the findings (5.61 ± 0.10 log_10_ cfu/cm^2^ for cutting boards and 6.43 ± 0.34 log_10_ cfu/cm^2^ for butchers' hands) from retail houses of Jigjiga town [35]. These variations of bacterial load might be due to the difference in meat processing, handling practices, and sanitary standard operating procedures along the meat production chain.

In most of the retail points, meats were seen on the ground and left to the mercies of the environment which can create an avenue for microbial pathogens to proliferate on it. These high bacterial loads could affect the average shelf life of the meat and increase the chances of spoilage.

### 3.3. Antibiotic Susceptibility Profile of *Staphylococcus aureus*

All the 55 positive samples of *S. aureus* isolates had undergone *in vitro* antibiotic susceptibility test on MHA medium by using a disc diffusion technique. The isolates were completely susceptible to ciprofloxacin followed by gentamycin (89.09%) (Table 1). This finding was agreed with the finding that all *S. aureus* isolates from meat samples in Addis Ababa were susceptible (100%) to ciprofloxacin [34]. This finding was also compatible with the previous research output from dairy farms and abattoirs in Addis Ababa that 97.7% of the isolates were susceptible to ciprofloxacin [9]. The reason for ciprofloxacin susceptibility of the isolates could be due to the fact that ciprofloxacin is a relatively expensive and newly introduced antibiotic as compared to the other common antibiotics [37].

The majority of the bacterial isolates (92.73%) were found to be resistant to the antibiotic penicillin and 74.5% of the isolates were resistant to cefoxitin. The present study was in harmony with the report indicating that 95.3% of the isolates were resistant to penicillin [9]. The reason for the high resistance of penicillin and other *β*-lactam antibiotics could be that they are the most commonly used antibiotics for the treatment of infection in humans and animals in Ethiopia [9].

According to the principles of CLSI, the susceptibility or resistance results of cefoxitin can be applied to methicillin [31]. In this research 74.5% of isolates of *S. aureus* were resistant to cefoxitin considered to be MRSA and out of the 41 cefoxitin resistant isolates, 36 (87.80%) were also resistant to penicillin. This could be due to the fact that the resistance of *S. aureus* to those drugs might be attributed to the presence of the *mecA* gene which has a low affinity for *β*-lactam antibiotics [39, 40].

**Figure 2:**
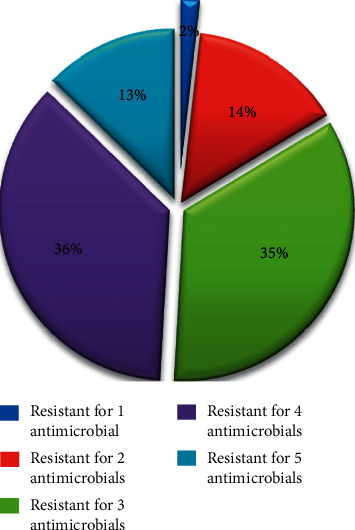
Drug-resistance patterns of *S. aureus* isolates from the raw beef sample in Bahir Dar city.

The present study revealed that about 98% of *S. aureus* isolates had high resistance to two or more drugs due to the fact that there is frequent irrational antimicrobial use and misuse behavior in the country [9] (Figure 2).

### 3.4. Multidrug-Resistance Profile of *S. aureus*

From the tested 55 isolates of *S. aureus*, 46 (83.64%) isolates showed MDR. Nineteen isolates were resistant to three antibiotics; similarly, twenty and seven isolates were resistant to four and five antibiotics, respectively (Table 2). The presence of MDR of *S. aureus* isolates in this study area indicates the possible significant risk of the resistant strain along the studied beef line.

## 4. Conclusion and Recommendations

This study revealed that the beef (ready-to-eat raw meat) in Bahir Dar city was highly contaminated with *S. aureus* and nearly two-thirds of contaminated samples had above the acceptable threshold level of bacterial load. To overcome this problem, providing food safety training for food handlers and safe meat handling (maintaining cold chain and cleanliness) is important. Of all tested antibiotics, all *S. aureus* isolates were totally susceptible to ciprofloxacin. On the other hand, the highest resistance was observed to penicillin followed by cefoxitin. Almost all of the isolates developed MDR and about half of them showed resistance to four or more classes of antibiotics suggesting wide distribution of MDR *S. aureus* stains from ready-to-eat beef existed. This may pose a risk to beef consumers due to the likelihood of food intoxication and antibiotic resistance problems. Therefore, appropriate *S. aureus* control strategies should be designed and implemented, staphylococcal infections due to *S. aureus* are better to be treated using the most potent antibiotics like ciprofloxacin and gentamycin with proper antimicrobial usage. Conducting further research regarding antibiotic resistance genes and enterotoxin genes on *S. aureus* is advisable.

## Figures and Tables

**Table 1:** Antibiotic susceptibility pattern of *S. aureus* isolates from the raw beef sample in Bahir Dar city (*N* = 55).

Antibiotic agent	Response of *S. aureus* for antibiotics *N* (%)		
	Susceptible	Intermediate	Resistant
**Chloramphenicol**	44 (80)	2 (3.63)	9 (16.36)
**Ciprofloxacin**	55 (100)	—	—
Cefoxitin	14 (25.45)	—	41(74.5)
Clindamycin	22 (40)	5 (9.09)	28 (50.9)
**Erythromycin**	40 (72.72)	8 (14.55)	7 (12.73)
**Gentamycin**	49 (89.09)	1 (1.81)	5 (9.09)
Penicillin	4 (7.27)	—	51 (92.73)
**Tetracycline**	16 (29.09)	4 (7.27)	35 (63.63)
Trimethoprim-sulfamethoxazole	39 (70.9)	3 (5.45)	13 (23.63)

**Table 2:** Multiple drug-resistance profile of *S. aureus* isolates from the raw beef sample in Bahir Dar city.

Number of antibiotics showing resistance	Number and frequency (%) of resistant isolates	Type of antibiotics
Three	19 (34.54%)	CAF + CLI + PEN CEF + CAF + PEN CEF + CLI + PEN (4) CEF + ERY + PEN CEF + ERY + TTC CEF + GEN + PEN CEF + PEN + TSX CEF + PEN + TTC (5) CLI + PEN + TSX PEN + TTC + TSX (3)

Four	20 (36.36)	CAF + CEF + CLI + PEN CAF + CEF + PEN + TTC (2) CAF + CLI + PEN + TTC CEF + CLI + ERY + TSX (2) CEF + CLI + PEN + TSX CEF + CLI + PEN + TTC (9) CEF + PEN + TTC + TSX (2) CLI + PEN + TTC + TSX GEN + PEN + TTC + TSX

Five	7 (12.73)	CAF + CEF + CLI + PEN + TTC (3) CEF + CLI + ERY + PEN + TTC CEF + CLI + GEN + PEN + TTC (2) CEF + GEN + PEN + TTC + TSX

CAF = chloramphenicol, CEF = cefoxitin, CLI = clindamycin, ERY = erythromycin, GEN = gentamycin, PEN = penicillin, TTC = tetracycline, TSX = trimethoprim-sulfamethoxazole.

## Data Availability

The raw datasets used and/or analyzed during the current study are available from the corresponding author on reasonable request.
